# The King Edward VII. Sanatorium

**Published:** 1906-07-07

**Authors:** 


### THE KING EDWARD VII. SANATORIUM.

THE King has approved of the following appointments in connection with the King Edward VII. Sanatorium at Midhurst.

CONSULTING STAFF.

Sir William Broadbent, Bart., K.C.V.O., F.R.S.
Sir Richard Douglas Powell, Bart., K.C.V.O.
Sir Felix Semon, K.C.V.O.
Sir Hermann Weber, M.D.
Sir Lauder Brunton, M.D., F.R.S.
C. Theodore Williams, Esq. M.V.O., M.D.
Professor Clifford Allbutt, M.D., F.R.S.
Professor Osler, M.D., F.R.S.
James Frederick Goodhart, Esq., M.D.
J. Kingston Fowler, Esq., M.D.
Percy Kidd, Esq., M.D.
William Bulloch, Esq., M.D.

EXECUTIVE COMMITTEE.

The Viscount Esher, G.C.V.O., K.C.B. (Chairman).
Sir Frederick Treves, Bart., G.C.V.O., C.B. (Deputy Chairman).
The Hon. Sidney Peel.
Sir Francis Laking, Bart., G.C.V.O.
Colonel Lascelles, M.V.O.
William James, Esq., D.L., J.P.
Rowland Bailey, Esq., M.V.O., I.S.O.
P. Horton-Smith Hartley, Esq., M.V.O., M.D. (Hon. Sec.).

LADY VISITORS.

Mrs. William James.
Miss Ethel McCaul, R.R.C.

